# Nitric Oxide (NO) Improves Wheat Growth under Dehydration Conditions by Regulating Phytohormone Levels and Induction of the Expression of the *TADHN* Dehydrin Gene

**DOI:** 10.3390/plants12234051

**Published:** 2023-12-01

**Authors:** Chulpan Allagulova, Azamat Avalbaev, Alsu Lubyanova, Anton Plotnikov, Ruslan Yuldashev, Oksana Lastochkina

**Affiliations:** Institute of Biochemistry and Genetics—Subdivision of the Ufa Federal Research Centre of the Russian Academy of Sciences, Prospect Oktyabrya 71, 450054 Ufa, Russia; avalbaev@yahoo.com (A.A.); lubyanova555@mail.ru (A.L.); plotnikow87@mail.ru (A.P.); oksanaibg@gmail.com (O.L.)

**Keywords:** *Triticum aestivum* L., nitric oxide, dehydration, transpiration intensity, relative water content, phytohormones, MDA, electrolyte leakage, dehydrins

## Abstract

Nitric oxide (NO) is a universal signaling molecule with important regulatory functions in the plant’s life cycle and adaptation to a wide spectrum of environmental stresses including drought. The effect of pre-sowing seed treatment with the donor of NO sodium nitroprusside (SNP, 200 μM) on wheat *Triticum aestivum* L. plants subjected to dehydration (PEG-8000, 12%) was investigated. SNP pretreatment stimulated germination and seedling growth in normal conditions and protected them under dehydration. These effects were confirmed by percentage of seed germination, changes in fresh and dry weight of 5–6-day-old seedlings, as well as by seedlings’ linear dimensions, visual appearance, and mitotic index of the root apical meristem. Assessment of the transpiration intensity (TI) and relative water content (RWC) showed that SNP pretreatment helped to maintain the water status of seedlings subjected to dehydration stress. The data obtained by enzyme-linked immunosorbent assay (ELISA) suggested that the positive effects of SNP may be due to its influence on the phytohormonal system. SNP pretreatment induced an increase in the level of indolylacetic acid (IAA) and especially cytokinins (CK), while essential changes in ABA content were not detected. Water deficiency caused a substantial increase in ABA content and a decrease in the levels of CK and IAA. Pre-sowing SNP treatment decreased stress-induced fluctuations in the content of all studied phytohormones. Using reverse-transcription PCR (RT-PCR), we obtained data on the increase in expression of the *TADHN* dehydrin gene in SNP-pretreated seedlings under normal and, especially, under dehydration conditions. These findings may indicate the participation of dehydrins in NO-induced defense reactions in wheat plants under water stress. Furthermore, exogenous NO had a stabilizing effect on membrane cellular structures, as evidenced by the reduction of electrolyte leakage (EL) levels and malondialdehyde (MDA) content in dehydrated wheat seedlings under the influence of pre-sowing SNP treatment.

## 1. Introduction

Drought is one of the most widespread abiotic stress factors, with detrimental effects on metabolic reactions and physiological processes throughout the plant life cycle [1,2,3]. The drought’s impact is especially destructive at the initial stages of ontogenesis during seed germination and the seedling emergence, which determine all subsequent growth and development and, ultimately, the plant’s productivity [4]. Various agrochemicals are widely used in practice to reduce crop losses. However, such compounds can pose a serious threat to the environment and human health when applied in excessive amounts. Therefore, one of the most important tasks of modern phytobiology is the search of safe and environmentally friendly approaches in improving stress tolerance and plant productivity. Pre-sowing seed treatment with biologically active compounds and plant growth regulators seems to be a promising method [5,6,7]. NO is an important signaling molecule involved in the regulation of fundamental metabolic processes at all the stages of the plant life cycle. It is essential in seed germination, vegetative growth, the cell cycle, tissue differentiation, root architecture formation, the establishment of symbiotic interactions, flowering, and fruit ripening [3,8,9,10]. Much interest is taken in NO due to its participation in the plant responses to a wide range of stress factors, including drought. Enhanced endogenous NO production has been detected in different plant species under drought conditions [11,12,13]. In addition, there is evidence of increased drought tolerance of plants under an exogenous NO application in a gaseous form or as its donors. The positive effects of NO are expressed in improved germination, vegetative growth, normalization of the water regime, maintenance of photosynthetic reactions, and reduction in the level of oxidative damage [2,3,10,13,14]. The functional activity of NO is realized in close interaction with the phytohormonal system. In particular, during seed germination, it affects the ratios of ABA, ethylene, and gibberellins concentrations, stimulating the dormancy breaking and seedling emergence [15]. NO is involved in auxin-dependent growth activation of lateral and adventitious roots and acts as a signaling intermediate in ABA-controlled stomatal movements [16,17,18].

Data on drought mitigation in different plant species under exogenous NO treatment suggest that it can be practically applied to improve the stress tolerance and productivity of crops [7,19,20,21,22,23]. This requires a deep understanding of the mechanisms underlying the biological activity of NO, which, at the molecular level, are realized through its interaction with numerous intracellular targets: reactive oxygen species (ROS), polyunsaturated fatty acids, peptides, etc. When interacting with proteins, NO leads to post-translational modifications (PTMs), such as S-nitrosylation, tyrosine nitration, and metal nitrosylation, which cause conformational rearrangements of protein molecules and changes in their functional activity [24]. Thus, NO can trigger transcription factors involved in the stress responses, with subsequent activation of the gene expression of defense proteins [25,26].

Among the most numerous dehydration-inducible genes are dehydrins, encoding LEA (Late Embryogenesis Abundant) proteins of group II, which accumulate in the seeds at late stages of their maturation during natural desiccation. These proteins are characterized by high hydrophilicity and structural plasticity and are essential for maintaining the embryo structures’ viability. Dehydrins also accumulate in vegetative plant tissues under water deficit conditions and exert various protective functions related to their chaperone, ion-binding, and antioxidant properties [27,28]. The regulation of dehydrin expression is carried out with the participation of ABA, which is not surprising, since it controls protective programs during plant dehydration [29]. The transcription activity of dehydrin genes and the accumulation of their protein products can be modulated by other signaling molecules, such as salicylic acid, jasmonates, and brassinosteroids, as was previously demonstrated in wheat plants [30,31,32]. However, the involvement of dehydrins in the protective effects of NO on plants under dehydration has been very poorly studied [3,27,28].

The present work is devoted to the investigation of the effect of pre-sowing seed treatment of wheat *Triticum aestivum* L. with the SNP at the concentration of 200 μM on germination, subsequent growth, transpiration intensity, relative water content (RWC), the state of the hormonal system, and the integrity of membrane cell structures of wheat seedlings under dehydration conditions simulated by treatment with PEG-8000. Considering the fact that dehydrins contribute significantly to the plant tolerance to water deficit conditions, a comparative analysis of expression of the *TADHN* dehydrin gene in wheat seedlings, pretreated with SNP and subjected to PEG-induced dehydration, was performed.

## 2. Results

### 2.1. Effect of SNP Pretreatment on Wheat Seed Germination and Seedling Growth under Dehydration Conditions

**Figure 1:**
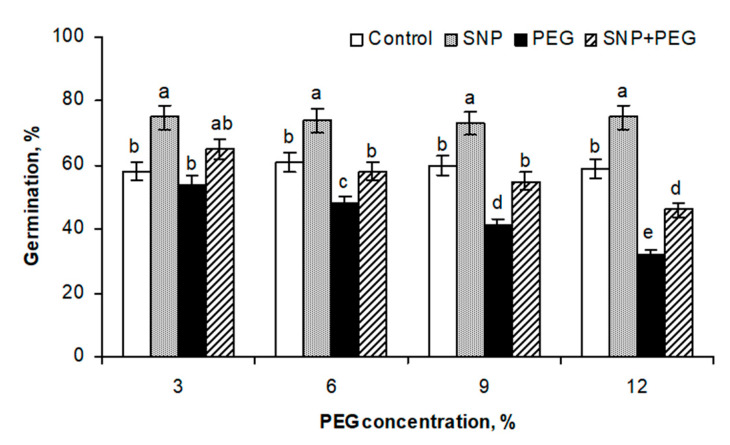
Effect of seed pretreatment with 200 µM SNP on germination of the soft wheat (*Triticum aestivum* L.) under dehydration conditions induced by the presence of PEG-8000 in the growth medium in increasing concentrations (3, 6, 9, and 12%). Data are given as mean values and their standard errors from three independent experiments. Distinct letters indicate that means are different at *p* < 0.05 (ANOVA, LSD test).

**Figure 2:**
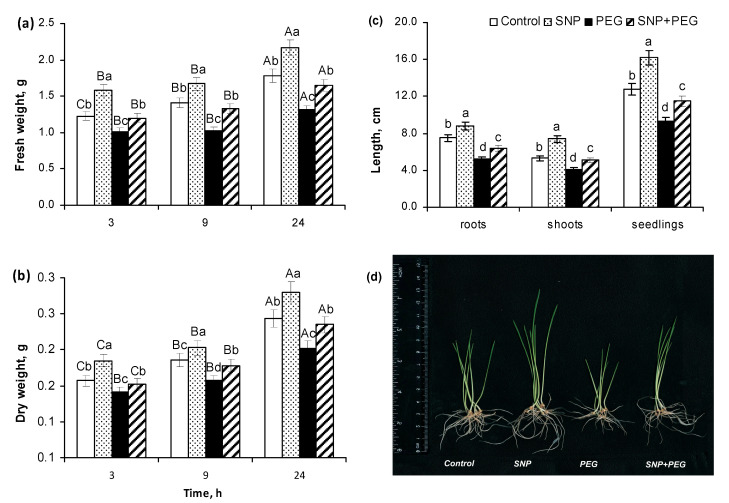
Effect of the seed pretreatment with 200 µM SNP on the growth of wheat seedlings, subjected to dehydration caused by treatment with PEG-8000 (12%) for 3, 9, and 24 h: (**a**) changes in the fresh weight of 5–6-day-old seedlings; (**b**) changes in the dry weight of 5–6-day-old seedlings; (**c**) linear parameters of roots, shoots, and whole 6-day-old seedlings; (**d**) visual appearance of 6-day-old wheat plants. Data are given as mean values and their standard errors from three independent experiments. Different lowercase letters indicate significant differences between treatments in a given comparison group: (**a**,**b**) time point; (**c**) roots, shoots, and whole seedlings at *p* < 0.05 (ANOVA, LSD test). Distinct capital letters indicate significant differences for the same treatment at different time points at *p* < 0.05 (ANOVA, LSD test).

**Figure 3:**
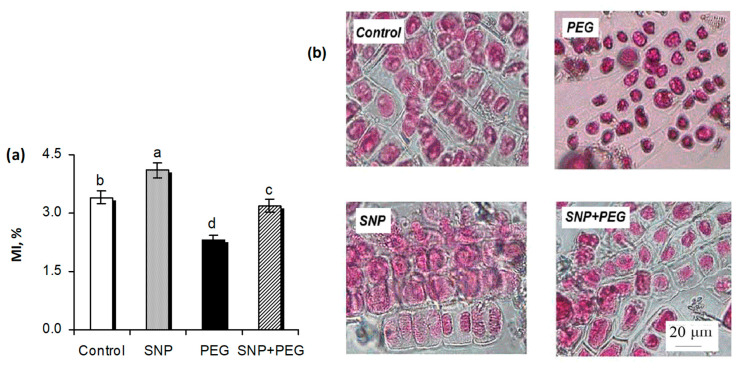
Effect of the seed pretreatment with 200 µM SNP on mitotic index (**a**) and cell division (**b**) of the root tips of 6-day-old wheat seedlings, subjected to dehydration (PEG 12%, 24 h). The pictures present pressed preparations of the roots’ apical part, stained with acetocarmine, visualized with fluorescence scanning microscope Biozero BZ-8100E (Keyence Co., Osaka, Japan). Scale bar = 20 µm. Data are given as mean values and their standard errors from three independent experiments. Distinct letters upper of the columns state that means are different at *p* < 0.05 (ANOVA, LSD test).

The influence of pre-sowing SNP treatment on wheat growth intensity under normal and dehydration conditions was assessed by the following indicators: seed germination (Figure 1), fresh weight (FW), dry weight (DW), seedlings’ linear parameters and visual appearance (Figure 2), and the mitotic index (MI) of the apical meristem of roots (Figure 3).

Soaking seeds prior to sowing in the 200 µM SNP solution for 3 h stimulated their germination under normal conditions, increasing seedling emergence by about 15% relative to the control level (Figure 1). Germination was suppressed under stress treatment. The inhibitory effect was already observed upon exposure to 3% PEG, although the seed germination level in this case was still comparable to the control values. Significant inhibition of germination was observed under the influence of 6% PEG, which became more severe as the osmotic concentration in the medium increased to 9% and 12%. Seed germination levels in these experiment variants decreased by 13%, 19%, and 27%, respectively. SNP pretreatment alleviated the harmful effect of dehydration on the germination of wheat seeds. Moreover, under mild stress (PEG 3%), the stimulatory effect of NO treatment on the level of seedling emergence was maintained. SNP pretreatment was helpful in preventing the inhibitory effect of 6% and 9% PEG and significantly mitigated its effect at a concentration of 12% (Figure 1).

Pre-sowing SNP treatment had a stimulating and protective effect on the subsequent vegetative growth of wheat seedlings under normal and dehydration conditions, respectively (Figure 2). The SNP pretreatment itself contributed to an increase in the fresh weight of seedlings by 21% and their dry weight by 15% to the sixth day of the experiment, as well as an increase in the linear sizes of roots, shoots, and whole seedlings by 16, 39, and 30%, respectively, to the same time period. The exposure to dehydration (PEG, 12%) for 3, 9, and 24 h resulted in 17%, 21%, and 26% losses in fresh weight and 10%, 16%, and 21% losses in the dry weight of seedlings, respectively (Figure 2a,b). The impact of dehydration for 24 h resulted in the reduction of the linear size of roots, shoots, and whole seedlings by 29%, 23%, and 27%, respectively (Figure 2c), which was evident from the visual appearance of the wheat plants (Figure 2d). SNP-pretreated and stress-subjected seedlings were characterized by maintaining the values of fresh and dry weight, as well as linear sizes at the values close to the control levels (Figure 2).

An important component of plant growth is the process of cell division in the apical root meristem. Pre-sowing SNP treatment promoted the root growth of wheat seedlings, as indicated by the increase of the mitotic index (MI) by more than 20% in comparison with SNP-untreated plants (Figure 3a), as well as representative images which clearly demonstrate the activation of cell division in the root tips of the wheat seedlings (Figure 3b). Analyzing the light microscopy data, it can be seen that a significant number of cells in the control samples were in the state of interphase, as evidenced by the presence of well-defined nuclear and clear cytoplasm surrounded by a membrane and cell wall. At the same time, in the control variant, cells with increased nuclear sizes and more intense granular staining in their centers are also distinguished, which may indicate the transition of the mitotic cycle to the metaphase stage (Figure 3b). In SNP-pretreated samples, actively dividing cells containing enlarged nuclei with dark granular staining in the center are clearly distinguishable, which may result from equatorial plate formation at the metaphase stage during mitosis. In these samples, the cells are not yet divided but contain two nuclei each, which corresponds to the telophase stage of mitotic division. The formation of a membranous septum can be seen in the equatorial region of one of the dividing cells. Thus, these data clearly demonstrate the stimulative effect of seed SNP-pretreatment on subsequent cell division in the root tips of 6-day-old wheat seedlings under normal conditions (Figure 3b). Stress exposure for 24 h decreased the MI value by almost 25%, which was reflected in the inhibition of cell division, which was registered microscopically by the reduction of cell size, compacted nuclei, and increased intensity of their staining, which most likely arose due to cell dehydration. 

SNP treatment contributed to the maintenance of the MI value and cell division process in roots of wheat seedlings under the influence of water deficit, which could be judged by the recovery of cell size and the character of their staining (Figure 3a,b). The obtained data indicate the effectiveness of pre-sowing seed treatment with 200 μM SNP for 3 h in stimulating the germination and vegetative growth of wheat plants under normal conditions and in their further protection if water stress occurs.

### 2.2. Effect of SNP Pretreatment on TI and RWC of Wheat Seedlings Subjected to Dehydration

**Figure 4:**
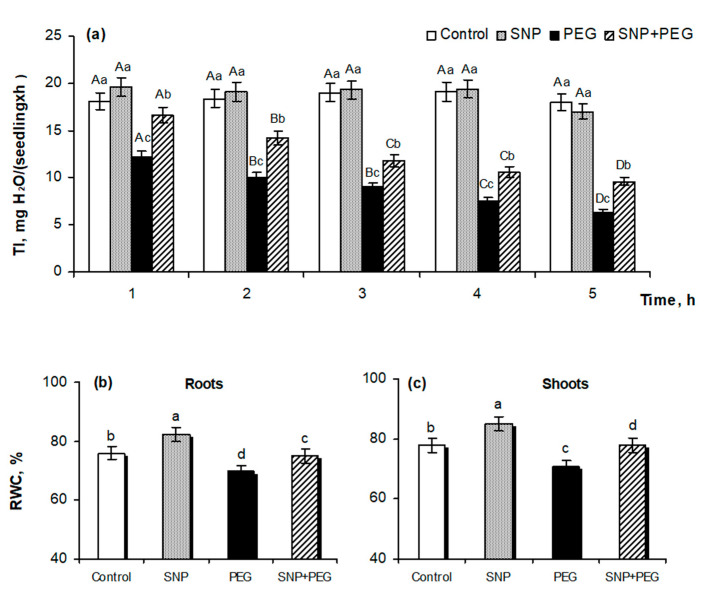
The effect of the seed pretreatment with 200 µM SNP on water regime parameters of 5-day-old wheat seedlings, subjected to dehydration, induced by 12% PEG treatment during 5 h: (**a**) transpiration intensity (TI) of leaves; (**b**) relative water content (RWC) of roots; (**c**) RWC of shoots. Data are given as mean values and their standard errors from three independent experiments. Lowercase letters indicate significant differences between treatments at *p* < 0.05 (ANOVA, LSD test) at the same time point. Capital letters indicate significant differences for the same treatment at different time points at *p* < 0.05 (ANOVA, LSD test).

SNP seed treatment had no significant effect on the transpiration process under normal growing conditions in wheat seedlings, in which values of transpiration intensity (TI) were close to the control levels (Figure 4a). Dehydration had a strong inhibitory effect on the transpiration process. Exposure to 12% PEG for 1 h resulted in a 38% reduction of TI, and it decreased further with each hour of stress treatment. Thus, by 5 h into the experiment, the TI in the stressed plants was three-fold weaker than in the control samples (Figure 4a). NO treatment mitigated the negative effect of dehydration on the transpiration process, as evidenced by higher TI values in SNP-treated samples compared with SNP-untreated ones under PEG-induced dehydration (Figure 4a). When the water content of the wheat seedlings was assessed, it was found that SNP pretreatment resulted in about a 6–7% increase in the levels of relative water content (RWC) of roots and shoots. Seedlings’ exposure to dehydration reduced the RWC values in shoots by about 10% and roots by 7% of the control level. SNP pretreatment favored the maintenance of RWC in roots and shoots at a level close to that of the control (Figure 4b).

### 2.3. Effect of SNP Pretreatment on Hormonal Balance of Wheat Seedlings Subjected to Dehydration

**Figure 5:**
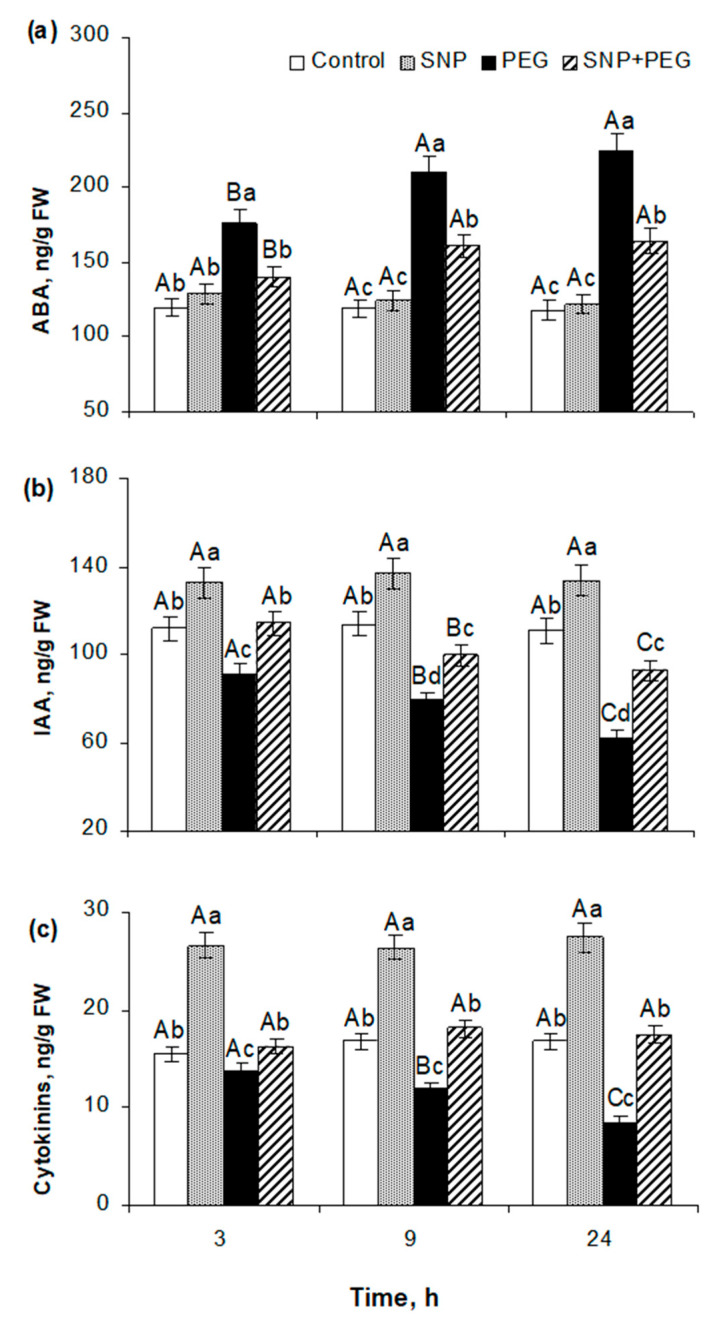
Effect of the seed pretreatment with 200 µM SNP on the content of ABA (**a**), IAA (**b**), and cytokinins (**c**) in 5–6-day-old wheat seedlings, subjected to dehydration caused by treatment with PEG-8000 (12%) for 3, 9, and 24 h. Data are given as mean values and their standard errors from three independent experiments. Lowercase letters indicate significant differences between treatments at *p* < 0.05 (ANOVA, LSD test) at the same time point. Capital letters indicate significant differences for the same treatment at different time points at *p* < 0.05 (ANOVA, LSD test).

Pre-sowing SNP seed treatment had a significant effect on the hormonal system of wheat seedlings as judged by changes in the abscisic acid (ABA), indolylacetic acid (IAA), and cytokinin (CK) content. In 5–6-day-old SNP-pretreated seedlings under normal growing conditions, in the absence of any significant shifts in ABA content, an increase in the concentration of IAA and CK by approximately 20% and more than 50%, respectively, was revealed. Their enhanced levels were kept in the seedlings throughout the experiment (Figure 5a–c). Under the influence of 12% PEG, ABA content increased about 47%, 78%, and 91% by 3, 9, and 24 h of stress exposure, while levels of auxins and cytokinins decreased approximately 18%, 30%, and 44% and 11%, 28%, and 49% after the same time periods, respectively (Figure 5). Pretreatment with the NO donor mitigated the sharp stress-induced fluctuations in the phytohormone content. In SNP-pretreated plants, ABA accumulation under stress conditions was about 20-25% lower compared with SNP-untreated plants, although it exceeded the control values by about 19%, 38%, and 40% after 3, 9, and 24 h, respectively (Figure 5a). The content of IAA in SNP-pretreated plants was sustained at higher levels in contrast to those of SNP-untreated ones when subjected to dehydration (Figure 5b). IAA concentrations in SNP-treated seedlings were comparable to control values after 3 h of dehydration and decreased only after 9 and 24 h of stress treatment by 12% and 16%, respectively (Figure 5b). SNP seed pretreatment was beneficial in preventing the stress-induced reduction of the cytokinin content in 5–6-day-old wheat plants (Figure 5c).

### 2.4. Expression of TADHN Dehydrin Gene

**Figure 6:**
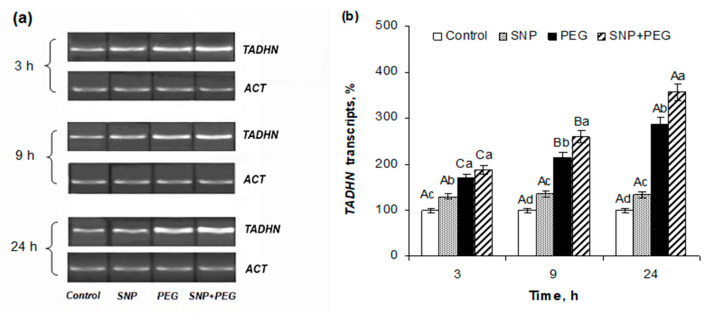
Effect of the seed pretreatment with 200 µM SNP on the expressional activity of *TADHN* dehydrin gene in the 5–6-day-old wheat seedlings, subjected to 12% PEG-8000 during 3, 9, and 24 h. (**a**) Semi-quantitative RT-PCR analysis of the accumulation of *TADHN* dehydrin transcripts; (**b**) data of densitometric analysis of *TADHN* gene transcript accumulation relative to the expression of the *ACT* gene, represented as percentages of the control levels. Data are given as mean values and their standard errors from three independent experiments. Lowercase letters indicate significant differences between treatments at *p* < 0.05 (ANOVA, LSD test) at the same time point. Capital letters indicate significant differences for the same treatment at different time points at *p* < 0.05 (ANOVA, LSD test).

SNP seed pretreatment resulted in the activation of *TADHN* dehydrin gene expression in 5–6-day-old seedlings, which was manifested by an increase in the level of its transcript accumulation by about 25–30% compared to that of the control SNP-untreated samples (Figure 6). Seedlings’ exposure to 12% PEG for 3, 9, and 24 h induced approximately 1.5-, 2-, and 3-fold increases of the *TADHN* gene transcript content. SNP pretreatment further increased the dehydration-induced expression of the *TADHN* dehydrin gene by about 50% so that at the end of the experiment (24 h), the level of dehydrin gene transcript accumulation reached more than 3.5-fold values relative to those of the control (Figure 6a,b).

### 2.5. MDA Accumulation and Electrolyte Leakage

**Figure 7:**
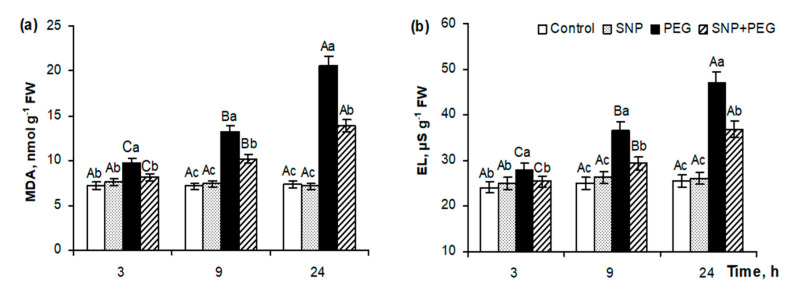
Effect of the seed pretreatment with 200 µM SNP on the MDA production (**a**) and electrolyte leakage level (**b**) in the 5–6-day-old wheat seedlings, subjected to 12% PEG-8000 for 3, 9, and 24 h. Data are given as mean values and their standard errors from three independent experiments. Lowercase letters indicate significant differences between treatments at *p* < 0.05 (ANOVA, LSD test) at the same time point. Capital letters indicate significant differences for the same treatment at different time points at *p* < 0.05 (ANOVA, LSD test).

Under normal conditions, pre-sowing SNP treatment did not induce changes in the production of malondialdehyde (MDA) and electrolyte leakage (EL) levels in 5–6-day-old seedlings (Figure 7). Dehydration, modulated by 12% PEG, had a strong damaging effect on cell membrane structures, inducing almost 2- and 3-fold increases in MDA concentrations by 9 and 24 h of stress treatment, as well as 1.5- and 2-fold increases of the EL levels from seedling tissues to the same time points. SNP pretreatment reduced the stress-induced MDA accumulation and EL levels by nearly 30% and 20%, respectively, after 24 h of the experiment, indicating the cytoprotective effect of NO on wheat plants under water deficit conditions (Figure 7).

## 3. Discussion

Drought is one of the most widespread and unpredictable stress factors, negatively affecting all aspects of plant metabolism and development and significantly limiting the productivity of different crops, including wheat [1]. The regulation of drought tolerance is carried out with the participation of the plant signaling system, an important component of which is NO. Its endogenous content in plants becomes much higher under water deficit conditions [2,11,13]. In addition, there is evidence of increased drought resistance of plants under exogenous application of NO in the form of gas or its donors, among which the most popular is SNP [2]. In this work, the effects of the pretreatment of wheat (*Triticum aestivum* L.) seeds with 200 µM SNP for 3 h on germination, subsequent seedling growth, their transpiration rate, relative water content, hormone balance, and expression of the *TADHN* dehydrin gene under normal conditions and under exposure to PEG-induced dehydration were analyzed.

It was found that SNP pretreatment had a stimulating and protective effect on the wheat seeds’ germination under normal and water deficit conditions (Figure 1), which is consistent with the literature data. NO-induced stimulation of seed germination has been demonstrated in *A. thaliana*, barley, lettuce, and other plant species [3,33,34]. Under the influence of NO treatment, apple embryos were found dormancy breaking [35]. Supplementation of 500 µM SNP or SNAP to the growth medium increased the germination potential of chickpea seeds due to the regulation of respiration, as was judged by oxygen consumption [36]. The germination-stimulating effect may be also due to the NO-induced increase in β-amylase activity, which leads to the enhancing of polysaccharide utilization. At the same time, NO is able to activate the pentose phosphate pathway of glucose catabolism by increasing the level of NADPH oxidation, which may contribute to the stimulation of the seed germination process in normal conditions and its protection under stress [3,8,37]. Evidence of NO’s ability to stimulate and protect the germination processes is of great practical importance. In this regard, it is worth mentioning that nitrates and nitrites have long been used to enhance seed germination, the stimulating effect of which may be due to the enhancement of endogenous NO production, which is carried out by reductive nitrite-dependent mechanisms [19]. The practical application of gaseous NO, as well as its donors and NO-releasing nanoparticles for seed priming for the purpose of inducing the dormancy breaking with a following increase in crop germination is also discussed in the literature [7,8,19].

We obtained data on the effectiveness of pre-sowing SNP seed treatment on the growth stimulation of wheat seedlings in normal conditions and their protection under water stress (Figure 2). SNP pretreatment increased the fresh and dry weight as well as the linear dimensions of 5–6-day old seedlings in normal conditions and reduced the negative effects of PEG-induced dehydration on the analyzed growth parameters (Figure 2a–c), which was clearly reflected in the visual appearance of the wheat plants (Figure 2d). The positive effect of NO on growth processes has been demonstrated in other works on examples of different cultivated and wild plant species [3,21,22,38,39,40]. In particular, *Atnoa1* mutant lines of *A. thaliana* with reduced endogenous NO production are characterized by severe growth inhibition and defective root development [38]. Spraying soybean plants (*Glycine max*) with 100 μM SNP improved their growth under normal conditions and under water stress, as evidenced by the data of their biomass increasing [39]. Pretreatment of *Physalis angulata* plants with SNP at concentrations of 50–100 μM had a positive effect on their growth and photosynthetic activity under soil drought [41]. Incubation of the root tips of 3-day-old maize seedlings in the presence of various NO donors such as SNP, S-nitrosoglutathione (GSNO), sodium nitrite, and nitrocysteine stimulated their elongation [40]. SNP treatment had an influence on the formation of the root system of tomato (*Lycopersicon esculentum*) and cucumber (*Cucumis sativus*) plants, increasing their branching by enhancing the growth of lateral and adventitious roots [42,43]. In tomato root sections, an increase in the expression activity of the cyclin genes *CYCD 3;1* and *CYCA 2;1* was observed under the influence of SNP treatment, indicating the involvement of NO in the regulation of the cell cycle [44]. This is also evidenced by the obtained data on the mitotic index (MI), which is an indicator of the cell division rate, as well as by the data of a light microscopic analysis of roots’ apical meristem (Figure 3a,b).

The establishment of drought tolerance in plants is highly dependent on their ability to maintain water status and limit water loss. In our experiments, it was shown that stress treatment (12% PEG) caused serious disturbances in the water balance of wheat, which were manifested in a significant decrease in the TI indices of leaves and RWC of roots and shoots of 5-day-old seedlings (Figure 4). SNP seed pretreatment had no significant effect on the transpiration process under normal conditions, and, as judged from the data of root and shoot RWC, contributed to a slight increase in the water uptake by wheat plants. This enhanced water uptake may contribute to the stimulation of growth processes upon exogenous NO application (Figure 2 and Figure 3) with the active participation of auxins and cytokinins, the increased levels of which were recorded in SNP-pretreated seedlings (Figure 5b,c). Under the influence of SNP pretreatment, compensation of the harmful effect of dehydration on the water balance parameters of wheat seedlings was observed. TI and RWC levels in SNP-pretreated samples under exposure to dehydration were more closely related to the control values in contrast to the SNP-untreated ones. These results are in agreement with the literature data. For example, 20-day-old *Brassica juncea* plants incubated in the presence of 100 µM SNP for 4 days maintained their indices of RWC, transpiration rate, and stomatal conductance under water stress (PEG, 10%) [14]. SNP treatment alleviated drought-induced decreases of leaf RWC in 7-day-old wheat plants [20]. Exogenous SNP treatment of lettuce (*Lactuca sativa* L.) improved photosynthesis by increasing chlorophyll synthesis; activated antioxidant enzymes, protecting lettuce plants against oxidative damage; and increased irrigation water consumption, thus increasing the yield under soil drought [23]. Thus, our results confirm the important role of NO in maintaining the water status of plants under drought, which is realized at physiological, biochemical and molecular levels through the modulation of stomatal movements; the increased synthesis of low-molecular-weight osmolytes: proline, glycine-betaine, and sugars; as well as through the changes in the gene expression activity which encode enzymes catalyzing the metabolism of osmoprotectants [2,3,4,13,14,17,20,45,46,47].

The fact that various physiological processes, in particular, germination, vegetative growth, and water metabolism, are regulated by phytohormones under normal or stress conditions [48,49] suggests that the growth-stimulating and protective effect of NO on wheat plants is due to the influence on their hormonal system. The analysis of changes in phytohormone content showed that pre-sowing SNP treatment had a noticeable effect on the hormonal system of wheat seedlings, in which, on the background of no significant changes in the amount of ABA, approximately a 20% increase in the level of IAA content and a more than 50% increase in the concentration of cytokinins were registered (Figure 5a–c). These data may indicate that the growth-stimulating effect of the NO donor may be associated with increased levels of accumulation of auxins and cytokinins, which belong to the recognized activators of plant metabolic activity [48,50]. PEG-induced dehydration caused a severe hormonal imbalance in wheat seedlings, associated with a significant increase in ABA concentration and a fall in CK and auxin content (Figure 5a–c). Pretreatment with NO donor favored the normalization of seedlings’ hormonal balance under dehydration conditions, which was expressed as a decrease in stress-induced accumulation of ABA and a weaker reduction in IAA concentration. Special attention should be paid to the fact that SNP pretreatment prevented a stress-induced decrease in the cytokinin concentration, which may contribute significantly to the realization of the protective effect of SNP, since hormones of cytokinin nature are characterized by the properties not only of growth stimulators, but also of inducers of plant stress resistance [49,50].

The stress-protective effect of NO at the molecular level is achieved by inducing transcription factors followed by activating the gene expression of defensive proteins [2,3,4,10]. A special role in protecting cellular structures from dehydration-induced damage belongs to dehydrins, which are well-known as group II of the LEA (late embryogenesis abundant) protein family [28,29,51]. They are accumulated in maturing seeds during the phase of their natural dehydration and play an important role in maintaining the viability of embryonic structures. Accumulation of dehydrins is also observed in the tissues of vegetative plants under abiotic stresses which cause cell dehydration, particularly under drought. The particular physicochemical properties such as intrinsic structural disorder, the presence of a conserved K-segment forming an amphiphilic α-helix, hydrophilicity, and a high polar amino acid content determine the chaperone, osmoprotective, and antioxidant properties of dehydrins, which allow them to perform multiple protective functions under water deficit conditions [28,29,32,51]. The obtained data on SNP-induced improvement of wheat tolerance to dehydration suggest that dehydrins may contribute to the protective action of NO. To test this hypothesis, a comparative analysis of the transcriptional activity of the *TADHN* dehydrin gene [52] in SNP-pretreated and untreated wheat seedlings under normal and water stress conditions was performed. Reverse-transcription–PCR analysis showed that pre-sowing SNP seed treatment promoted the subsequent activation of the dehydrin gene in 5–6-day-old seedlings in the absence of stress, which was exhibited by an approximately 30% increase in the accumulation level of *TADHN* gene transcripts (Figure 6). Exposure to stress for 3, 9, and 24 h caused nearly 1.5-, 2-, and 3-fold increases in the levels of dehydrin’s transcript accumulation in wheat seedlings, which is not surprising since water stress induces the synthesis of these key osmoprotectants [29,32,51,53]. SNP-pretreated seedlings were characterized by an additional increase in the expression activity of the *TADHN* dehydrin gene under dehydration, so that after 24 h of stress, the levels of accumulation of its transcripts were 50% higher compared to SNP-untreated and stressed plants and more than 3.5-fold higher than in the control (Figure 6). There are only limited data on the participation of dehydrins in NO-controlled plant defense responses under water deficit conditions. Thus, enhanced expression of *Y_2_SK*, *Y_2_K*, and *SK_2_* dehydrins in drought-exposed white clover (*Trifolium repens*) plants was observed under the treatment with spermidine belonging to the polyamines, which are one of the sources of oxidative NO production in photosynthetic organisms [3,54,55]. In transgenic rice plants overexpressing the rat *nNOS* gene with increased endogenous NO production, an enhancement in the expression activity of the transcription factors OsDREB2A and OsDREB2B, which are involved in the regulation of expression of many drought-related genes, including dehydrins, was observed, as well as an increase in the gene expression of the *OsLEA3* protein, which belongs to the family of late embryogenesis abundant proteins [25]. The increase in endogenous NO content in the resurrection plants *Haberlea rhodopensis* under drought conditions was accompanied by the accumulation of dehydrin proteins, which was detected by Western blot analysis [56]. Thus, the presented literature data in conjunction with the results obtained in this work may indicate the participation of dehydrins in NO-controlled plant defense responses against the harmful effects of drought.

It is well-known that the rapid nonspecific plant response to environmental stresses is a sharp increase in the generation of reactive oxygen species (ROS), which induce oxidative damage of biopolymers and membrane lipids, affecting the configuration and functions of cellular structures. NO can reduce the level of oxidative stress by interacting with ROS or by activating the antioxidant system (AOS) of plants [3,9,10,13,20]. Therefore, it could be expected that SNP pretreatment would help neutralize ROS and mitigate the damaging effects of drought on membrane structures’ integrity, as can be judged by accumulation of MDA, which is an indicator of lipid peroxidation, and the level of electrolyte leakage. Our experiments showed that pretreatment of seeds with 200 μM SNP for 3 h had no effect on MDA content and electrolyte leakage in 6-day-old wheat seedlings (Figure 7a,b), which confirms the fact that such treatment is not stressful but is favorable, judging from the growth-stimulating effect (Figure 1 and Figure 2). Dehydration modulated by 12% PEG induced significant damage of the membrane structures, as evidenced by a 3-fold increase in MDA production and an about 2-fold increase in electrolyte leakage by 24 h of stress exposure (Figure 7a,b). SNP-pretreated plants were characterized by significantly lower levels of MDA accumulation and exoosmosis of electrolytes under dehydration, indicating the involvement of NO in plant protection against oxidative stress. The NO molecule is characterized by a free-radical nature, which explains its ability to be involved in the regulation of plant redox homeostasis, which is realized by direct interaction with ROS or by activation of enzymatic and non-enzymatic antioxidants. In particular, when NO interacts with superoxide-anion radical (O_2_^●−^), peroxynitrite (ONOO^−^) is formed, which belongs to the class of active nitrogen forms and is characterized by high reactivity. However, the level of its toxicity is much lower than that of O_2_^●−^ or H_2_O_2_, so its formation is considered as a direct NO-induced mitigation of oxidative burst during stress impact [34,57]. An important contribution to the protective action of NO can be made by its ability to stimulate plant AOS, inducing the formation of enzymatic and non-enzymatic antioxidants and increasing their activity under environmental stress factors, including drought.

## 4. Materials and Methods

### 4.1. Seed Material, SNP Pretreatment

Seeds of spring soft wheat *Triticum aestivum* L., variety Ekada-70, were collected from the Chishminsky Breeding station (Bashkortostan, Russian Federation). Seeds were sterilized in 96% ethanol for 1 min, washed thoroughly with running water, drained of excess moisture with filter paper, and soaked for 3 h in a solution of NO donor sodium nitroprusside (SNP) at a concentration of 200 μM. Samples soaked in distilled water served as control.

### 4.2. Assessment of Seed Germination

To evaluate the protective effect of NO on wheat germination under drought conditions, SNP-treated and untreated seeds were sown in Petri dishes containing a disk of filter paper moistened with 10 mL of water (control) or PEG-8000 solutions at concentrations of 3%, 6%, 9%, and 12%. Samples were placed in the thermostat TS-1/80 SPU (Smolensk, Russia) with a temperature of 21 °C, and seeds were germinated in the dark for 3 days. Germination was determined by the percentage ratio of the number of germinated seeds to the total number of sown seeds.

### 4.3. Seedling Growth

SNP-pretreated and untreated seeds were sown in Petri dishes with filter paper moistened with 10 mL of water and germinated at 20-21°C, 16 h photoperiod, and illumination of 180 µmol m^−2^ s^−1^. Then, 4-day-old samples were transferred into glasses of up to 1 L, 30 seedlings in each glass containing 40 mL of 10% Hoagland-Arnon solution, and incubated for 24 h to stabilize the growth process. After that, 5-day-old seedlings were subjected to dehydration by using PEG-8000 at a concentration of 12% for 3, 9, and 24 h. Growth intensity was judged by changes in fresh and dry weight of 5–6-day-old seedlings; linear sizes of roots, shoots, and whole seedlings; their visual appearance; and mitotic index of root apical meristem, which were evaluated after 24 h of stress treatment. For subsequent analysis of phytohormone content, MDA production, and accumulation of *TADHN* dehydrin gene transcripts, plant material was fixed in liquid nitrogen and stored at −70 °C.

### 4.4. Determination of Mitotic Index (MI)

To determine the MI, the apical part of seedling roots together with the root cap was fixed for 1.5 h in a 1:3 mixture of acetic acid and ethanol. After fixation, plant material was washed for 30 min in running tap water and then treated for 1 h with a mixture of 5% pectinase (EC 3.2.1.15) (ICN, Costa Mesa, CA, USA) and 5% cellulase (EC 3.2.1.4) (ICN, USA); enzyme treatment was carried out at 37 °C. The pressed preparations were stained with acetocarmine made in 45% acetic acid solution. MI of apical root meristem cells was counted as the sum of cells in prophase, metaphase, anaphase, and telophase stages, expressed as the percent of the total number of counted cells [58]. In each experimental variant, 5000 cells were analyzed using an AxioImager M1 microscope (Carl Zeiss, Jena, Germany) equipped with an N-Achroplan 63×/0.85 M27 objective. Images were documented with a fluorescence scanning microscope (Biozero BZ-8100E, Keyence Co., Osaka, Japan) using a 63×/0.85 M27 objective and ImageJ software (https://imagej.net/Fiji (accessed on 20 October 2020)).

### 4.5. Determination of the Transpiration Intensity (TI)

To assess the intensity of transpiration, which was carried out using the gravimetric method, 10 seedlings were used per experimental variant. The 5-day-old seedlings were placed into the small glasses containing 10 mL of 10% Hoagland–Arnon solution or a mixture of 10% Hoagland–Arnon solution with 12% PEG-8000. Then, the glasses were covered with aluminum foil to prevent evaporation. The glasses were weighed every hour from 1 h to 5 h of stress treatment. The transpiration rate was calculated by subtracting the value of the final weight of the sample from the value of the initial weight, followed by division by the number of seedlings [59].

### 4.6. Determination of the Relative Water Content (RWC)

RWC analysis was performed using the same samples in which TI was evaluated. For calculation of the RWC, it was necessary to determine the fresh weight (FW), turgor weight (TW), and dry weight (DW) of roots and shoots [60]. For this purpose, 5-day-old seedlings were dissected into roots and shoots, and their FW was immediately determined. Then, the samples were placed in vessels with distilled water, covered with lids, incubated for 24 h in the dark, and weighed to determine the values of TW. After that, samples were dried at 70 °C for 48 h and their DW values was determined. The relative water content (RWC) was calculated by the formula:

RWC = [(FW − DW)/(TW − DW)] × 100%.

### 4.7. Extraction and Immunoassay of Phytohormones

The content of free IAA, ABA, and cytokinins in one plant sample was determined by enzyme-linked immunosorbent assay (ELISA) using rabbit antibodies specific to each of these hormones and peroxidase-labeled anti-rabbit antibodies. The extraction of phytohormones (ABA, IAA, and cytokinins) from one plant sample was carried out according to the method described in detail earlier [48]. Samples from 10 seedlings were grounded into powder in liquid nitrogen, and phytohormones were extracted in 80% ethanol at 4 °C. After 16 h, the samples were centrifuged for 15 min at 12,000× *g*, and the supernatant was evaporated in a stream of air to an aqueous residue. An aliquot of the aqueous residue was used for the subsequent determination of the total content of zeatin derivatives (zeatin, zeatin-riboside, and nucleotide) immunopositive to serum obtained against zeatin riboside. ABA and IAA were extracted with sulfuric ether from the remaining aqueous extract and subjected to immunoassay using polyclonal rabbit antibodies to ABA and IAA.

### 4.8. Relative Level of TADHN Gene Transcripts Accumulation

The transcriptional activity of the *TADHN* dehydrin gene was investigated using a semi-quantitative RT-PCR analysis, as was described in detail previously [32]. Total RNA, which was extracted by Trizol reagent, was purified from genomic DNA contaminants using DNAse (Thermo Fisher Scientific Inc., Waltham, MA, USA). The reaction of the single-stranded cDNA construction was performed using M-MLV reverse transcriptase (Promega, Madison, WI, USA) in 10 µL of a mixture containing 2 µg of total RNA, 3 pmol of oligo-(dT)10-16-primers in reaction buffer (50 mM Tris-HCl, pH 8.4, 10 mM DTT, 36.5 nmol of MgCl_2_, 75 mM KCl). The obtained cDNA samples were amplified using gene-specific primers (Table 1) [52]. Two reference genes were used in the experiments: actin (ACT) (GenBank accession No. EF592180) and CJ705892 (GenBank accession No. CJ705892), which exhibited high expression stability in wheat seedlings under short-term drought conditions [61,62]. Sequences of the primers are given in Table 1. Figure 6 shows the data of *TADHN* dehydrin gene expression relative to the ACT gene.

### 4.9. Measurements of MDA Content and Electrolyte Leakage

Changes in MDA content and electrolyte leakage levels served as indicators of lipid peroxidation and membrane permeability, respectively [63]. To determine MDA content, the sample of plant material (0.5 g) was grounded in distilled water in a ratio of 1:6. Then, samples were mixed with an equal volume of 20% trichloroacetic acid, incubated in a water bath (98 °C, 30 min), cooled, and centrifuged for 10 min at 12,000× *g* at 4 °C. An aliquot of the supernatant was mixed with an equal volume of a 0.5% solution of thiobarbituric acid in 20% trichloroacetic acid. The MDA content, expressed in nmol g^−1^ FW, was calculated from the optical density of the samples, which was measured at 532 and 600 nm using spectrophotometer SmartSpecTM Plus (Bio Rad). To determine the electrolyte leakage, the samples of plant material (1 g) were cut into equal sections of about 1 mm and placed into the small glasses with 20 mL distilled water. Samples were stirred on a shaker (ES-20, Biosan, Rīga, Latvia) at 140 rpm, 35 °C for 1.5 h, filtered, and used for electrical conductivity measuring using a conductometer/TDS meter HI 8633 (HANNA Instruments, Catania, Italy). Electrolyte leakage levels were judged from the obtained conductivity values expressed in µS g^−1^ FW [63].

### 4.10. Statistical Analysis

All experiments were performed in at least three or four biological and analytical replicates. Statistical analysis of the obtained data was carried out by ANOVA, SPSS version 19.0 for Windows (SPSS Inc., Chicago, IL, USA). The significance of differences was evaluated using the LSD test at *p* < 0.05.

## 5. Conclusions

The data obtained in this work indicate that SNP, which is a donor of NO, displayed the properties of a growth stimulator and inductor of wheat plants’ resistance to dehydration when used at a concentration of 200 μM for a 3 h seed treatment. This was clearly demonstrated in a comparative analysis of seed germination as well as when assessing the fresh and dry weights, linear dimensions, and cell division rates of SNP-pretreated wheat seedlings subjected to PEG-induced dehydration. The beneficial effect of NO donor on wheat plants under normal and water deficit conditions may be attributed to its participation in the regulation of water regime, as indicated by RWC and TI data, and achieved through its interaction with the phytohormonal system, judging from the changes in the content of ABA, IAA and CK. SNP treatment of seeds caused the accumulation of auxins and cytokinins in the seedlings, both of them being known activators of plant metabolic activity, which can explain the pronounced growth-stimulating effect of NO. At the same time, these hormones are also involved in the regulation of stress responses in plants. In addition, SNP-pretreated plants were characterized by a decrease in the amplitude of stress-induced fluctuations in the content of all studied phytohormones. The data obtained in this work on the increase in the expression of the *TADHN* dehydrin gene in seedlings under the influence of SNP pretreatment under normal and especially under stress conditions may indicate the dehydrins’ involvement in the protective effects of NO on wheat plants under dehydration. The data on the decline in MDA production and electrolyte leakage levels in dehydrated wheat seedlings under the influence of SNP pretreatment support the ability of NO to participate in plant defense against oxidative stress under drought.

## Figures and Tables

**Table 1:** Characteristics of the primers.

Gene Name	GenBank Accession Number	Primer Sequence (5′ → 3′)	PCR-Product Length (bp)	Reference
*TADHN* (*Triticum aestivum* dehydrin)	AY574032	F: CATCGATGAGAACGGTGAGGTG R: TGTCCATGATCTTGCCCAGTAGG	390	[49]
*ACT* (*Triticum aestivum* mRNA for actin)	AB181991	F: GGAGAAGCTCGCTTACGTG R: GGGCACCTGAACCTTTCTGA	136	[61,62]
EST CJ705892	CJ705892	F: GCCTCAGTGGTAGGAGCATT R: TTCAGCAAATGCGGTGGTTG	116	[62]

PCR products were separated in 1.5% agarose gels, stained 10 min in 0.5 µg/mL ethidium bromide, and viewed in the transilluminator Gel Doc XR (Bio Rad Laboratories, Hercules, CA, USA). The results were photo-documented using the Gel Camera System (Bio Rad Laboratories).

## Data Availability

Data are contained within the article.
